# Assessment of Destructive and Nondestructive Analysis for GGBS Based Geopolymer Concrete and Its Statistical Analysis

**DOI:** 10.3390/polym14153132

**Published:** 2022-07-31

**Authors:** Fatheali A. Shilar, Sharanabasava V. Ganachari, Veerabhadragouda B. Patil, Syed Javed, T M Yunus Khan, Rahmath Ulla Baig

**Affiliations:** 1 Department of Civil Engineering, Jain College of Engineering, Belagavi 590014, Karnataka, India; shilarone@gmail.com; 2 Department of Chemistry, School of Advanced Science, KLE Technological University, Hubballi 580031, Karnataka, India; 3 Institute of Energetic Materials, Faculty of Chemical Technology, University of Pardubice, 53210 Pardubice, Czech Republic; iamveerabhadraa@gmail.com; 4 Department of Mechanical Engineering, College of Engineering, King Khalid University, Abha 61421, Saudi Arabia; jjaffer@kku.edu.sas (S.J.); yunus.tatagar@gmail.com (T.M.Y.K.); 5 Research Center for Advanced Materials Science (RCAMS), King Khalid University, Abha 61413, Saudi Arabia; 6 Department of Industrial Engineering, College of Engineering, King Khalid University, Abha 61421, Saudi Arabia; rub786@gmail.com

**Keywords:** geopolymer concrete, compressive strength, alkaline to binder ratio, casting

## Abstract

Geopolymer is the alternative to current construction material trends. In this paper, an attempt is made to produce a sustainable construction composite material using geopolymer. Ground granulated blast furnace slag (GGBS)-based geopolymer concrete was prepared and tested for different alkaline to binder ratios (A/B). The effect of various temperatures on compressive strength properties was assessed. The cubes were exposed to temperature ranging from 50 to 70 °C for a duration ranging from 2 to 10 h, and the compressive strength of the specimens was analyzed for destructive and non-destructive analysis and tested for 7, 28, and 90 days. The obtained compressive strength (CS) results were analyzed employing the probability plot (PP) curve, distribution overview curve (DOC), probability density function (PDF), Weibull, survival, and hazard function curve. Maximum compressive strength was achieved for the temperature of 70 °C and an A/B of 0.45 for destructive tests and non-destructive tests with 44.6 MPa and 43.56 MPa, respectively, on 90 days of testing. The survival and hazard function curves showed incremental distribution characteristics for 28 and 90 days of testing results with a probability factor ranging from 0.8 to 1.0.

## 1. Introduction

GGBS is the one of the industrial wastes which shows excellent replacement potential for FA in the manufacturing of geopolymers concrete. Si^4+^ and Al^3+^ are more highly soluble in sodium hydroxide (NaOH) agents than in potassium hydroxide (KOH) activator agents [1,2]. NaOH, when added to GGBS, silica, alumina, and other small ions, initiates dissolution. A strong alkaline agent leads to the dissolution alumino-silicates reactive material, and free SiO_4_ and AlO_4_ tetrahedral structures formation takes place [3,4]. As a result, the rate of geopolymerization accelerates, which makes it possible for the composite to develop high compressive strength (CS) at temperatures (TE) ranging from 40 to 95 °C [5,6,7]. When the amount of GGBS content is increased from 0 to 30%, it results in the increment of CS up to certain limit for ambient curing [8,9,10]. The CS of 15.2 MPa was achieved at ambient temperature curing when tested at day 3, with a lower sodium hydroxide concentration [11,12]. When FA-based GPC was activated with sodium silicate and sodium hydroxide, tested at days 3, a CS of 10 MPa was observed [13,14,15].

Si and Al interacted with −OH^−^ ion, leading to development of calcium-silicate-hydrate in a GPC, which results in a significant strength development in matrix. The amount of NaOH as an activator agent when it gets completely dissolved leads to a significant impact on the amount of matrix left in the pore space. A higher Si and Al content in the matrix leads to the acceleration of the geopolymerization rate and less residual alkali. The activator dissolves the silica, resulting in an increase in the structural strength [16]. The activator interacts with less silica content during polymerization than acute necessary for reaction, resulting in low levels of residual alkali in the pore space [17,18,19,20]. The mechanistic differences between hydroxide and silica in polymer formation linked to the changes that occur during gel precipitation, with hydroxide-activator gels forming mostly in the presence of FA [21,22]. 

As per the recent literature, when waste glass powder is used as a binder in the making of GPC, results show that glass powder, which is rich in Si and Al in its chemical composition, enhances the rate of geopolymerization and leads to the development of a polysilicate bond, causing the incrementation of CS [23]. The development of sodium-aluminosilicate-hydrate (N-A-S-H) gel is attributed to the endurance of GPC during the geopolymerization process at a micro level. The Si and Al are the major contents in glass waste, and these ions get dissolved by the activator agent resulting in the formation of N-A-S-H gel. The development of aluminosilicate oligomers is caused by interactions between small dissolved ions and silicate originated from activating agent [1,23]. N-A-S-H gel develops over time, eventually becoming solid crystals that contribute to geopolymerized bonding. According to past research, the strength development of cementitious composites depends on the curing condition and the curing temperature. GPC specimens casted with A/B: 0.60 and beyond this ratio result in the decline in CS, for the specimens exposed to the ideal curing temperature. The cracks were observed at ITZ zone after the specimens were being exposed to temperatures above 600 °C. GPC has a higher durability than OPC, and calcium concentration has a significant impact on the durability mechanism [2,23,24]. There is relatively less research that has been carried out on the temperature effect on the strength development of geopolymer. In particular, the temperature effect compared with the destructive and nondestructive testing on CS analysis is one such area of research. It was also observed from recent literature that the temperature effect plays vital role in the enhancement of geopolymerization [5,6,7,8,9,10,11,12,13,14,15,16,17,18,19,20,21,22,23,24,25].

The present study shows the feasibility of GGBS as a binder in preparation of GPC. Destructive (DT) and nondestructive testing (NDT) methods were used to analyze the GPC specimens which were prepared with different mix proportions. Attempts made to predict a concrete attribute from an NDT measurement may lead to significant uncertainty as a result of various variables influencing NDT results. The use of a rebound hammer to determine concrete compressive strength (CS) may cause variation in 5–10% in the CS values compared to destructive testing [4,5,25]. An attempt is made in this paper to produce high strength geopolymer using different ratios of A/B ranging from 0.30 to 0.75, when specimens are exposed from 50 to 70 °C, for the duration of 2 h to 10 h for 7, 28, and 90 days of testing. 

**Figure 1:**
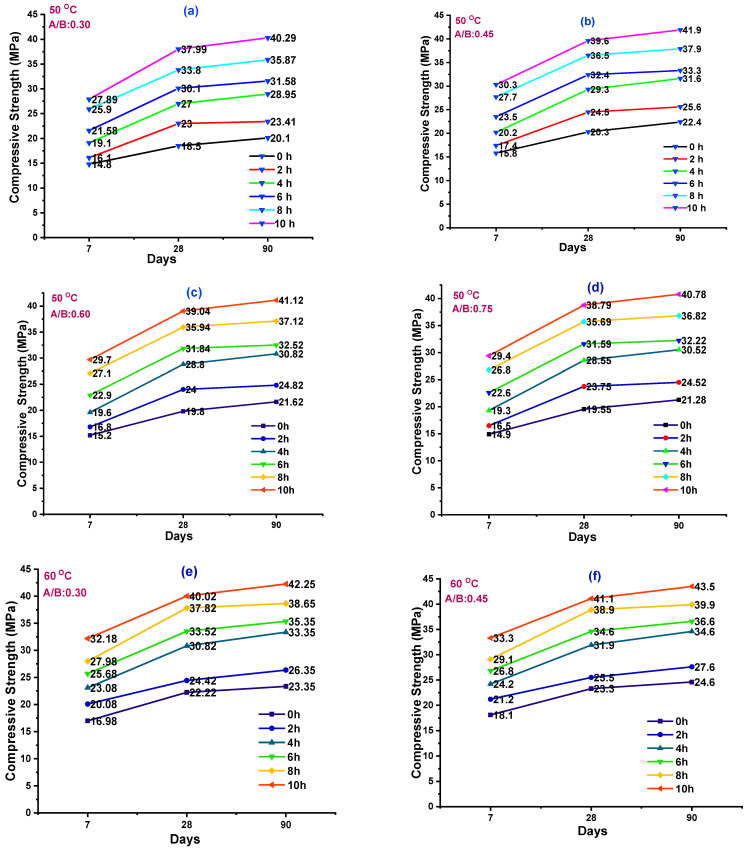

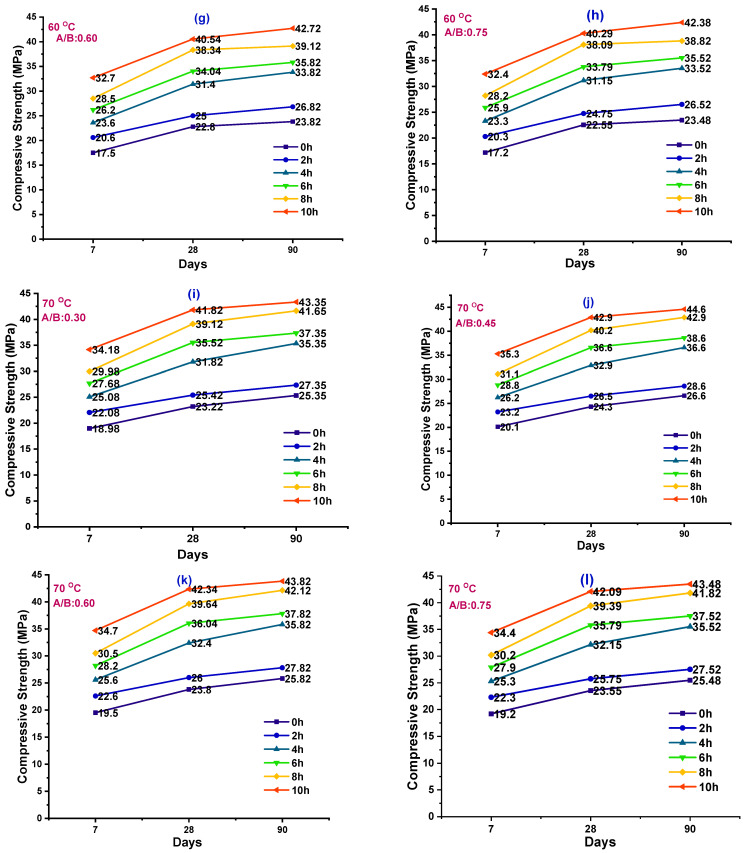
CS results of DT analysis tested at 7, 28, and 90 days. GPC specimens were cured at different temperatures: (**a**–**d**) cured at 50 °C, (**e**–**h**) cured at 60 °C, (**i**–**l**) cured at 70 °C.

Destructive testing results were represented in 12 different graphs (Figure 1a–l) for various temperatures such as 50, 60, and 70 °C. Rebound hammer equipment was utilized for NDT analysis. A set of four pairs of each comprised of 18 specimen cubes were prepared. These 18 cubes were cast with different A/B ratios of 0.30, 0.40, 0.60, and 0.75, and cured at 50 °C. Each cube was examined with a rebound hammer after 7, 28, and 90 days. The probability density function (PDF), distribution overview curve, Weibull, survival, and hazard function curves were used for the analysis of compressive strength results.

## 2. Materials and Methodology

### 2.1. Materials Characteristics

GGBS was used as a binder in the making of GPC, and the chemical compositions and physical attributes of GPC are given in Table 1 and Table 2. NaOH and Na_2_SiO_3_ were used as activator agents. For the current investigation, GGBS was purchased from Bellary, with as specific gravity of 2.88, a specific surface of 400 m^2^/kg, and a bulk density of 1100 kg/m^3^. These basic tests were carried out according to IS 12089 codes [26]. Testing of fine and coarse aggregate was carried out as per IS: 383 [27]. The aggregates were obtained from locally available stores; Mega chemicals, Hubballi, Karnataka, India, which provided NaOH and Na_2_SiO_3_. Both fine and coarse aggregate belong to Zone 2. Fine aggregate with a downsize of 4.75 mm has a specific gravity of 2.6, a fineness modulus of 3, and a water absorption of 1%. The coarse aggregate, with a downsize of 20 mm, has a specific gravity of 2.8, a fineness modulus of 7.0, and a water absorption of 1.12%, which was observed from experimentation. Distilled water was added during the preparation of the activator agent to maintain molar concentration of solution. 

### 2.2. Samples Preparation

Mix design was prepared referring to previous literature and IS 10262 codes [28]. Basic tests and analyses were performed on all GPC constituents. NaOH was in the form of a solid pellet, which was transformed to a liquid by adding a sufficient amount of water while keeping a molar concentration of 16 M. Each ingredient was weighed and dry-mixed consistently. The activator agent was prepared a day before specimen casting. GPC ingredients were mixed with the concrete mixer. Initially, the dry mix of binders was blended with activator agents. Geopolymer concrete was mixed in the concrete mixer for 5 to 10 min. The table vibrator was used to compact the concrete in mold. Each molds was vibrated for 5 min. Cubes were cured at room temperature, covering with gunny bags. Cubes molds of 150 × 150 × 150 mm in size were used for the study of destructive and nondestructive testing. Cube specimens were tested in compression testing machine after 7, 28, and 90 days of casting at 140 kg/cm^2^ per minute loading rate until they failed. Table 3 shows a mix proportion used in preparation of GPC. A total of 12 different specimens were cast, namely from G0 to G11. Initially, G0 to G2 specimens were cast with an A/B ratio of 0.30, G3 to G5 specimens with an A/B ratio of 0.45, G6 to G8 specimens with an A/B ratio of 0.60, and finally G9 to G11 compressed with an A/B ratio of 0.75. All 12 specimens were exposed to a temperature of 50 °C, and for the duration of 0 to 10 h, similar specimens were cast and exposed to a temperature of 60 and 70 °C.

### 2.3. Method of Analysis 

#### 2.3.1. Destructive and Nondestructive Testing

Compressive strength tests for destructive analysis were carried out as per IS 1199 [29] recommendations. A compression testing machine (CTM) was used for the destructive analysis (DT) of the cubes. CTM used for testing has a maximum capacity of 2000 KN. Specimens were tested in CTM after 7, 28, and 90 days of testing with the loading rate of 140 kg/cm^2^ per minute until they fail. The compressive strength was calculated by dividing the value of the force at which the yield to the cross-sectional area of the tested specimen appeared.

Non-destructive surface hardness techniques are noninvasive approaches used to assess material strength properties. Concrete surface hardness techniques are classified into two types: indentation methods and rebound methods. These approaches try to capitalize on empirical relationships between concrete strength qualities and surface hardness as evaluated by indentation or rebound. The conventional rebound hammer test is the most regularly used surface hardness technique. Schmidt, a Swiss engineer, invented the test in 1948, and it is now known as the Schmidt rebound hammer. The bounced hammer records a rebound number upon impact with the concrete surface, which provides an indicator of strength qualities by referencing proven empirical connections between concrete strength parameters (compressive and flexural) and the rebound number. Non-destructive testing (NDT) was carried out with the rebound hammer as per IS 13311(Part 2) [30] recommendations. The rebound hammer plunger presses on the specimen surface, and the spring-controlled mass rebounds, the extent of which is determined by the specimen surface hardness. The rebound number is noted from the rebound equipment, and the same number is referred to find the compressive strength using a calibration chart.

#### 2.3.2. Statistical Analysis of Tests

The results of destructive testing (DT) and non-destructive testing (NDT), probability plot (PP) curve, distribution overview curve (DOC), probability density function (PDF), Weibull, survival, and hazard function curve were plotted for different ratios of A/B and for testing at days 7, 28, and 90 using mini tab software. 

## 3. Analysis of Compressive Strength (CS)

### 3.1. Destructive test (DT)

Cubes of sizes 150 × 150 × 150 mm were used for DT analysis. A 18 cube specimens were prepared with an A/B ratio of 0.30, and all 18 cubes were exposed to a temperature of 50 °C for the duration of 0 to 10 h; the results are plotted in Figure 1a. Similarly, for different ratios of A/B such as 0.45, 0.60, and 0.75, the cubes were prepared and exposed to the temperature of 50 °C for the duration of 0 to 10 h, as referred to in Figure 1b–d. In a similar manner, different cubes were exposed to temperatures of 60 °C and 70 °C; the results are plotted in Figure 1e–h,i–l. Table 4 represents DT results for various temperature and A/B ratios.

#### 3.1.1. Effect of Temperature (50 °C) on GPC

GPC was prepared with GGBS as 100%. GGBS has a specific gravity of 2.88 along a specific surface of 400 m^2^/kg. After casting the cubes, they were kept in the laboratory under control condition for 1 h for hardening, and then they were kept in the oven under the required TE. After the cubes were oven cured, they were cooled at room TE (30 °C). A CS test was carried out as per IS 516 codes in the compression testing machine. GPM was cast for different TE exposures ranging from 0 h to 10 h for the duration of 2 h. CS results for GPM with an A/B ratio of 0.30, 0.45, 0.60, and 0.75 for 7, 28, and 90 days are shown in Figure 1a–l.

The CS results for the A/B ratio of 0.30 for 50 °C were in the range of 14.68–29.18 MPa, 19.22–38.52 MPa, and 21.5–40.65 MPa for 7, 28, and 90 days, respectively. The maximum CS values observed were 29.18, 38.52, and 40.65 MPa for the cubes which were exposed to the duration of 10 h, when tested at 7, 28, and 90 days, respectively, as shown in Figure 1a. For an A/B ratio of 0.45 for 50 °C, CS value was seen to range from 15.8 to 30.13 MPa, 20.3 to 39.6 MPa, and 22.4 to 41.9 MPa for 7, 28, and 90 days, respectively. The maximum CS values were seen to be 30.13, 39.6, and 41.9 MPa at 10 h tested for 7, 28, and 90 days, respectively, as shown in Figure 1b.

For an A/B ratio of 0.60 for 50 °C, the CS values ranged from 15.2 to 29.7 MPa, 19.8 to 39.04 MPa, and 21.62 to 41.12 MPa for 7, 28, and 90 days of testing. The maximum CS values were seen to be 29.7, 39.04, and 41.12 MPa at 10 h tested for 7, 28, and 90 days, respectively, as shown in Figure 1c. For an A/B ratio of 0.75 for 50 °C, the CS value was seen to range from 14.9 to 29.4 MPa, 19.55 to 38.79 MPa, and 21.28 to 40.78 MPa for 7, 28, and 90 days, respectively. The maximum CS value was seen as 29.4, 38.79, and 40.78 MPa at 10 h tested for 7, 28, and 90 days, respectively, as shown in Figure 1d.

The temperature for ambient and heat curing are 25 and 80 °C, respectively, and the mechanical property of GPC changes in an incremental way, accordingly. The sodium-silicate-to-sodium-hydroxide ratios and curing condition play a vital role in strength development [25,26,31]. The CS values of all the GPC specimens were found to be higher than their original CS values between 20 and 400 °C. According to previous research, the maximum temperature that can withstand before losing strength is 600 °C for GPC specimens. At 600 °C, the coarse aggregate of the GPC specimens had a crushing index of 7.7%. Thermal deterioration of the coarse aggregate induced cracking and spalling in the GPC specimens over 600 °C, lowering their CS. At all temperatures, GPH-A (oven-cured condition) had a larger percentage residual strength than GPH-H (ambient-cured condition), and GPC had a superior CS augmentation and less strength depreciation than heat-cured GPC [26,31].

#### 3.1.2. Effect of Temperature (60 °C) on GPC

Figure 1e–h represents the CS results; the specimens were prepared with an A/B ratio of 0.30 and subjected to a temperature of 60 °C. The CS values ranged from 16.96 to 32.18 MPa, 22.22 to 40.02 MPa, and 23.35 to 42.25 MPa for 7, 28, and 90 days, respectively. The maximum CS values were observed to be 32.18 MPa, 40.02 MPa, and 42.25 MPa when cubes were exposed to the temperature of 60 °C for the duration of 10 h and tested for 7, 28, and 90 days, respectively, as shown in Figure 1e. For an A/B ratio of 0.45 for 60 °C, the CS values were seen to range from 18.1 to 33.3 MPa, 23.3 to 41.1 MPa, and 24.6 to 43.5 MPa for 7, 28, and 90 days, respectively. The maximum CS values were seen to be 33.3, 41.1, and 43.5 MPa when cubes were exposed for the duration of 10 h and tested for 7, 28, and 90 days, respectively, as shown in Figure 1f.

For an A/B ratio of 0.60 for 60 °C, the CS values ranged from 17.5 to 32.7 MPa, 22.8 to 40.54 MPa, and 23.82 to 42.72 MPa for 7, 14, and 28 days, respectively. The maximum CS values were observed to be 32.7, 40.54, and 42.72 MPa at 10 h curing for 7, 28, and 90 days, respectively, as shown in Figure 1g. For an A/B ratio of 0.75 for 60 °C, the CS values ranged from 17.2 to 32.4 MPa, 22.55 to 40.29 MPa, and from 23.48 to 42.38 MPa for 7, 28, and 90 days. The maximum CS values were seen to be 32.4, 40.29, and 42.38 MPa at 10 h curing for 7, 28, and 90 days, respectively, as shown in Figure 1h.

The CS results vary with molar concentration of alkali activators and curing conditions. The CS increases as the molar concentration also increases; however, it decreases beyond an acceptable threshold in the oven-cured specimen. The maximum CS of oven-cured 14 M mix is 34.2 MPa after 56 days, while the ultimate CS of ambient cured 16 M mix is 25 MPa. The maximum CS for various molar ranges ranging from 8 to 16 M with a 2 M interval at 56 days for ambient curing is 13.9 to 25.0 MPa. For oven curing, CS ranges from 23.2 to 31.0 MPa for molar ranges ranging from 8 to 16 M with a 2 M interval. The CS varies with the Na_2_SiO_3_/NaOH (SS/SH) ratio and the curing conditions. The development of polycondensation between tetrahedral aluminosilicate gels might explain the improvement in CS. Chemical reactions take place in Al-Si materials in highly alkaline solution conditions, resulting in polymeric Si-O-Al-O bonds [26,32]. The alkali concentration has a crucial role in improving the polymerization process and strength growth. The alkali content and Na_2_O/Al_2_O_3_ ratio contribute more efficiently to the formation of the geopolymer phase. The pH increased as the molarity increased, which promotes the amorphous phase formation [3,26,32].

#### 3.1.3. Effect of Temperature (70 °C) on GPC

The experimental results of CS values for an A/B ratio of 0.30 for 70 °C are in the range of 18.98–34.18 MPa, 23.22–41.82 MPa, and 25.35–43.35 MPa for 7, 28, and 90 days. The maximum CS values were observed to be 34.18, 41.82, and 43.35 MPa at 10 h curing for 7, 28, and 90 days, respectively, as shown in Figure 1i. For an A/B ratio of 0.45 for 70 °C, the CS values are in the range of 24.3–42.9 MPa, 24.3–42.9 MPa, and 26.6–44.6 for 7, 28, and 90 days, respectively. The maximum CS values were observed to be 42.9, 43.4, and 44.6 MPa at 10 h curing for 7, 28, and 90 days, respectively, as shown in Figure 1j.

For an A/B ratio of 0.60 for 60 °C, the CS values were in the range of 19.5–34.7 MPa, 23.8–42.34 MPa, and 25.82–43.82 MPa for 7, 28, and 90 days, respectively. The maximum CS values were seen to be 34.7, 42.34, and 43.82 MPa at 10 h curing for 7, 28, and 90 days, respectively, as shown in Figure 1k. For an A/B ratio of 0.75 for 60 °C, the CS values ranged from 19.2 to 34.4 MPa, 23.55 to 42.09 MPa, and 25.48 to 43.48 MPa for 7, 28, and 90 days. The maximum CS values were observed to be 34.4, 42.09, and 43.48 MPa for 10 h, as shown in Figure 1l.

The CS of GGBS-based GPC treated both in the oven and steamed at 60 °C for 24 h is higher at an early stage than concretes cured under typical standard curing conditions [24,33]. Because of its quick hardening period, the geopolymer has a high early strength. The GPM may reach 70% of its full strength after 4 h of curing. The strength increased as the NaOH concentration increased, owing to the leaching out of silica and alumina with high NaOH concentrations and high Na_2_O/Al_2_O_3_ ratios [25,33]. The higher the NaOH concentration, the more Na ions there were in the solution, which was critical for geopolymerization since Na+ ions were utilized to balance the charges and produce the alumina-silicate networks [33]. In GGBS, calcium is a major component in its chemical composition, leading to the formation of compound glassy natured calcium-alumina-silicates, which in turn led to the enhancement of the geopolymerization process [34]. For GPC, the SS/SH was calculated as 2 and 2.5. On day 3, the greatest CS value was recorded for a Si/Al ratio of 2 with 30 MPa strength. Researchers experimented with FA as a binding material, varying the SS/SH ratio from 1.75 to 3 [35]. They concluded that at ambient temperature, the greatest CS was reached at a 2.5 ratio. Between 2.5 and 3, there was just a little rise in CS value. The influence of temperature as a curing condition in the Si/Al ratio was examined for exposure durations of 24 to 48 h at 60, 75, and 90 °C [32,35]. They found that a maximum CS value was attained with a Si/Al ratio of 2.5 and a temperature of 75 °C for 24 h. The greatest CS value for 14M with the oven-cured specimen is 35.7 MPa after 56 days. The highest CS value at 56 days with an ambient-cured specimen is 25.8 MPa [21,35].

When aluminosilicate solid binders react with an alkaline solution, they form a three-dimensional polymeric structure. The ultimate strength of the GPC is determined by the ratio (Si/Al) with the most commonly used materials ranging between 2 and 3.5. Geopolymerization processes involve activators such as sodium hydroxide (NaOH) or potassium hydroxide (KOH) and silicates as supplementary ionic molecules with a strong affinity core. The process by which an alkali-activated aluminosilicates binder hardens is the dissolution of Si and Al in the presence of NaOH [8,20,35]. The precipitation of calcium silicate or alumina hydrate is caused by the formation of NaOH. The clay mineral interacts with alkali to generate an aluminosilicate hydrate. Natural mineral polycondensation and hydroxylation with alkaline activation result in polymer material with a 3-D cross-linked polysialate chain. Polycondensation of polymeric precursors produces Si and Al ions, while polysialates produce Si–O–Al bonds. Since a more viscous activator agent lowers the quantity of unreacted GGBS particles in the matrix, a strong bond between silica and alumina ions forms. The Na_2_SiO_3_/NaOH ratio decreases, making Na_2_SiO_3_ less dense than NaOH, resulting in a reduction [5,36].

### 3.2. Non-Destructive Testing (DNT)

**Figure 2:**
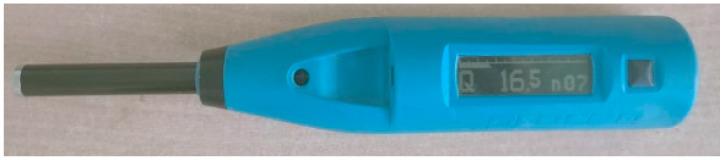
Rebound hammer equipment.

**Figure 3:**
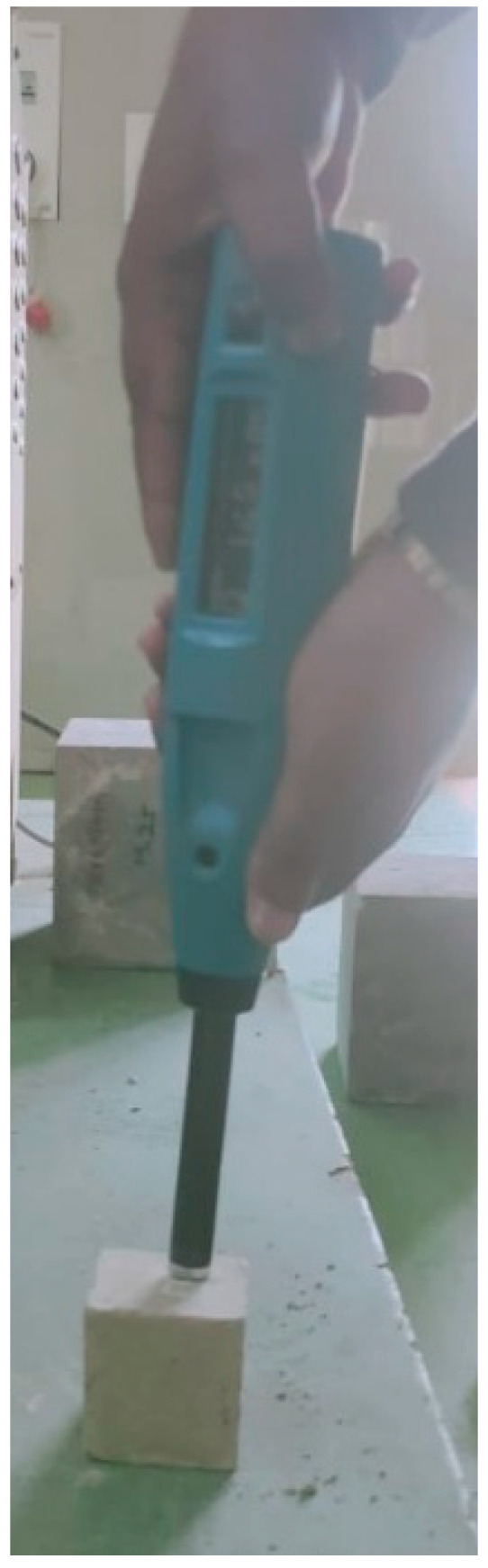
Calibration of rebound hammer equipment.

**Figure 4:**
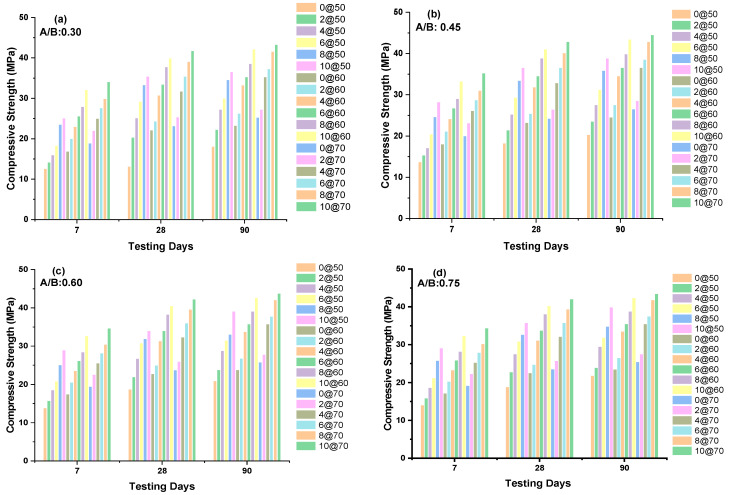
(**a**–**d**). The NDT test results for 7, 28, and 90 days of testing. (**a**) NDT tests at A/B: 0.30, (**b**) NDT tests at A/B: 0.45, (**c**) NDT tests at A/B: 0.60, (**d**) NDT tests at A/B: 0.75.

For NDT analysis, rebound hammer equipment was used. Figure 2 shows the rebound equipment and Figure 3 represents the calibration of the rebound equipment. A set of four pairs of 18 cubes were prepared; each pair of 18 cubes was casted with A/B ratios of 0.30, 0.40, 0.60, and 0.75, and the cubes were subjected to a curing temperature of 50 °C. Each cube was thermally cured for 0 to 10 h before testing with a rebound hammer at 7, 28, and 90 days. The NDT findings are shown in Figure 4. Similarly, 18 cubes were cast for four different A/B ratios and subjected to temperatures of 60 and 70 °C, as shown in Figure 4a–d. According to the findings of the experiments, when the A/B ratios increase, the CS value increases up to a certain point, beyond which the strength begins to decline. A/B ratios up to 0.45 exhibit the greatest increase in CS when compared to 0.30, 0.60, and 0.75 ratios. GPC specimens with a 10-h duration at 50 °C exhibit the highest CS value when compared to 0, 2, 4, and 6 h.

For an A/B ratio of 0.45, 10 at 50 (for 10 h of duration, the specimens were exposed to a temperature of 50 °C), this ratio was found to have % increment of CS values of 8%, 6.5%, 4.6%, and 3.2% compared with 0 at 50, 2 at 50, 4 at 50, and 6 at 50, respectively, at day 7. For an A/B ratio of 0.45, at day 28, 10 at 60 (for 10 h of duration, the specimens were exposed to a temperature of 60 °C), this ratio was found to have increments of CS values of 9%, 7.5%, 5.9%, and 3.8% compared with 0 at 60, 2 at 60, 4 at 60, and 6 at 60, respectively. For an A/B ratio of 0.45, at day 90, 10@70 (for 10 h of duration, the specimens were exposed to a temperature of 70 °C), this ratio (for 10 h at a temperature of 70 °C, exposed to GPC) was found to have increments of CS values of 6%, 4.3%, 3.1%, and 1.9% compared with 0 at 70, 2 at 70, 4 at 70, and 6 at 70, respectively. 

When oven curing and the A/B ratio of GPC increased, the CS values increased up to a specific level. A combination of sodium silicate and sodium hydroxide was utilized as an activator agent during the preparation of GPC [33,34,35]. Types of activators have a significant impact on polymerization acceleration. When an activator agent is added to binders, it promotes the Si and Al crystallization of the structure, which transforms the oligomer to monomer and accelerates the chemical process of geopolymerization in the matrix. When GGBS and FA were used as binders along with a superplasticizer, it enhanced the workability and the better development of strength compared with the mix without superplasticizer [14,31,32,37]. The influence of temperature as a curing condition in the A/B ratio was studied at 60, 75, and 90 °C for exposure durations ranging from 24 to 48 h [38,39]. It was discovered that a maximum CS value was attained with an A/B ratio of 0.35 for a temperature of 75 °C for a period of 24 h. The rate of hydration decreases when the molar increases from 8 M to 12 M, and this mix consist of GGBS up to 60% in the overall binder content. A/B ratios also play a vital role in strength development; a recent study observed that an A/B ratio of 0.45 shows excellent mechanical performances compared with 0.30 when FA is used as binder, curing under ambient condition [25,38,40,41]. At the micro level study, when GGBS is combined with a higher molar concentration, a weaker interfacial transition zone (ITZ) was observed followed by a hair-line crack appeared at aggregate and geopolymer paste [42,43].

After the activator agent at 20 °C and 40 °C, geopolymer dissolution and production of geopolymer precursors begins within the first 10 min. However, because more FA and GGBS may dissolve at high temperatures, greater temperatures are predicted to speed up reaction rates. At high temperatures, the dissolving process and the synthesis of geopolymer precursors can go on for longer. The viscosity of the samples is increased when more geopolymer precursors develop. As a result, the Si/Al molar ratio may have a significant influence on the mechanical and microstructure characteristics of geopolymeric materials when they are subjected to higher temperatures. Furthermore, previous research into the thermochemistry and thermal characteristics of fly ash used well-thought-out geopolymers based on fly ash with an objective for amorphous Si/Al ratios greater than two [15,27,29,42,43]. The amorphous content of fly ash, as well as the Si/Al ratio, influenced the reaction to thermal exposure, with higher ratios offering improved responses when exposed to temperatures of up to 100 °C. A lower Si/Al ratio in the manufacturing of geopolymers is used for enhanced heat resistance. The measurements of thermal volume shrinkage of samples after being exposed to higher temperatures kept track of significant reductions in shrinkage values above 20 wt. percent alumina additions. This was especially true above 80 °C, leading to the conclusion that the presence of alumina was beneficial in reducing thermal shrinkage and speeding up crystallization at a set temperature, as well as the extent of crystallization fillers, when combined with an Al plus Si-containing geopolymer based on metakaolin. The absence of inorganic type fillers in the microstructure might be due to a geopolymer gel covering or the particles of filler reacting in the geopolymerization kinetics, rendering them indistinguishable [5,6,8,30,43].

Metakaolin-based concrete was shown to have a higher strength than concrete containing fly ash, silica fume-incorporating concrete, and standard OPC-concrete up to 100 °C. The greater strength concretes quickly disintegrate after reaching 100 °C Despite having a superior initial strength increase, metakaolin-based concrete displayed the lowest final residual strength, indicating that it is particularly sensitive to a wider temperature range. There are several differences in the performance of concrete containing pozzolanic elements when exposed to high temperatures. Between 40 and 100 °C, there was strong stability and better early strength increases, followed by explicit decline and lower ultimate compressive strength than the reference [6,9,30,43].

## 4. Statistical Analysis of Tests

### 4.1. Probability Plot (PP)

**Figure 5:**
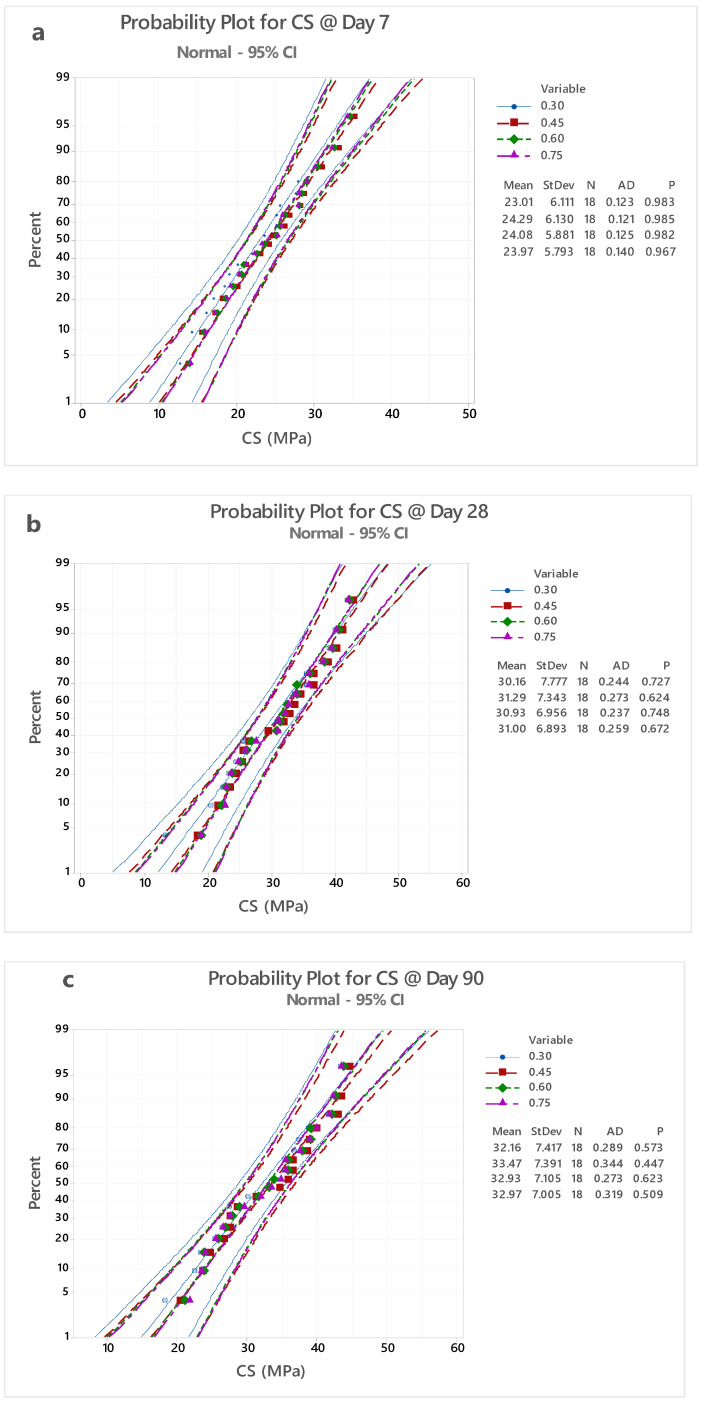
(**a**) Shows the PP curve for CS results at day 7; (**b**) shows the PP curve for CS results at day 28 and (**c**) shows the PP curve for CS results at day 90.

Geopolymer concrete was prepared with different proportions of A/B, such as 0.30, 0.45, 0.60, and 0.75, and the findings of the destructive results, such as compressive strength parameters of different A/B ratios were used for the computation of probability plot (PP). Figure 5 shows the PP analysis; here, an A/B ratio of 0.30 was found to have a maximum probability value of 0.983, and its PP indicates a regression value. In Figure 5, the probability plot shows the band which includes all the four different values of A/B. At percentages of 30 to 70, the band width is narrow but at percentages of 71 to 99 and 29 to 1 the band shows incremental width. The band at the central portion indicates that all A/B ratios are closely packed at 30 to 70 percent bands. When we compared the different A/B ratios with PP values, it can be observed that PP values were in the range of 0.967–0.983. 

Figure 5a–c shows the PP curve for CS results. PP curve was analyzed using mini tab software. GPC specimens with an A/B ratio of 0.30 testing after day 7 show a probability factor of 0.983, as seen in Figure 5a. It indicates 98.3% as the CS results line which varies linearly. Similarly, GPC specimens with an A/B ratio of 0.45, 0.60, and 0.75, tested after day 7, show a probability factor of 0.985, 0.982, and 0.967, respectively. It indicates 98.5%, 98.2%, and 96.7% is the CS results line which varies linearly. 

All four specimens had a deviation ratio ranging from 0.123 to 0.140. Under the curing temperature of 50 °C, CS values ranged from 13 MPa to 36 MPa. The factors that were considered for the preparation mix design and casting measure adopted for the preparation of GPC were 96 to 98% closer to accurate at 50 °C, for a testing period of 7 days, and PP was shown to be 0.98 to 0.96, specifying the factors that were considered for the preparation mix design and casting measure adopted for the preparation of GPC were 96 to 98% nearer to accurate.

After 28 days of testing, a GPC specimen with an A/B ratio of 0.30 shows a probability factor of 0.727, as seen in Figure 5b. The CS results line, which varies linearly, indicates 72.7%. Similarly, following day 28, GPC specimens with A/B ratios of 0.45, 0.60, and 0.75 show likelihood factors of 0.624, 0.748, and 0.672, respectively. The CS results line indicates 62.4%, 74.8%, and 67.2%, which fluctuates linearly. For all four specimens, the deviation ratio ranged from 0.244 to 0.259. Under the curing temperature of 60 °C, CS values ranged from 13 MPa to 44 MPa. For 60 °C, a 28-day testing period, and a PP of 0.62 to 0.74, it is stated that as the temperature was increased from 50 to 60, few specimens showed increments of CS value but the variation of strength of the GPC specimens tested at day 7 is almost double. Standard deviation at 28 days was found to be 1.5% times higher than at 7 days of testing. 

Figure 5 shows a probability factor of 0.573 for a GPC specimen with an A/B ratio of 0.30.The CS results line, which varies linearly, indicates 57.3 percent. After 28 days, the probability factors of 0.447, 0.623, and 0.509 were observed in GPC specimens with A/B ratios of 0.45, 0.60, and 0.75, respectively. The linear variations in the CS results line are 44.7%, 62.3%, and 50.9%. All four specimens had deviation ratios ranging from 0.289 to 0.319. At a curing temperature of 60 °C, the CS values ranged from 18 MPa to 45 MPa. When the temperature was elevated from 60 to 70 °C, the findings for 70 °C, a 90-day testing period, and a PP of 0.44 to 0.50 were obtained. When the temperature was elevated from 60 to 70 °C, at a 90-day testing period, and at a PP of 0.44 to 0.50, it was shown that the CS increased along with non-uniformity of incremental strength for various GPC specimens in spite of being cast under identical conditions [44,45]. Despite having superior initial strength increase, metakaolin-based concrete displayed the lowest final residual strength, indicating that it is particularly sensitive to a wider range of temperatures. There are several differences in the performance of concrete containing pozzolanic elements when exposed to high temperatures [46]. The good stability and larger initial strength improvements between 40 and 80 °C, followed by the explicit decline and lower ultimate CS than reference concrete, are often attributed [47,48,49,50,51,52,53]. The effect of disparity in relation to the Si/Al molar ratio on volume stability, mesoscale, strength endurance, or macroscale properties of metakaolin geopolymers has been identified. A geopolymer specimen with a Si/Al-1.75 ratio had a maximum compressive resistance of 6 MPa. It was discovered that, due to the increased amount of cracking and reduced residual compressive resistance, it is important to improve the macro-scale stability of GP with metakaolin in order to use it as a structural fireproof material [53,54,55,56,57,58,59].

### 4.2. Distribution Overview (DO) Plot

**Figure 6:**
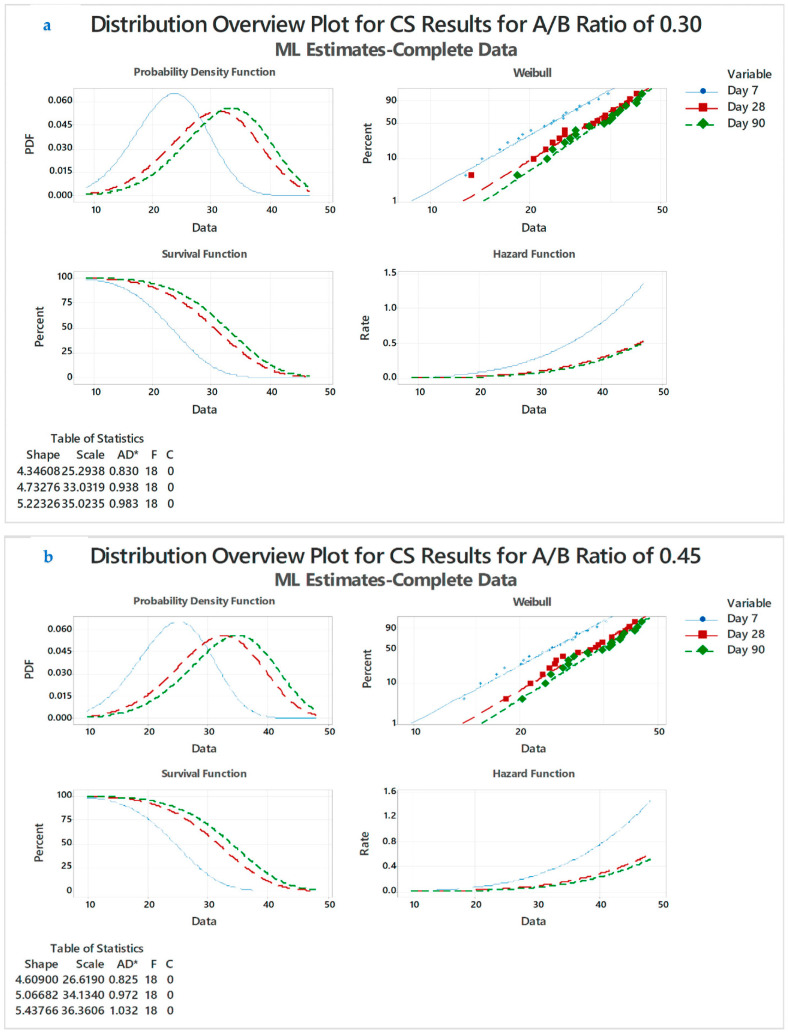

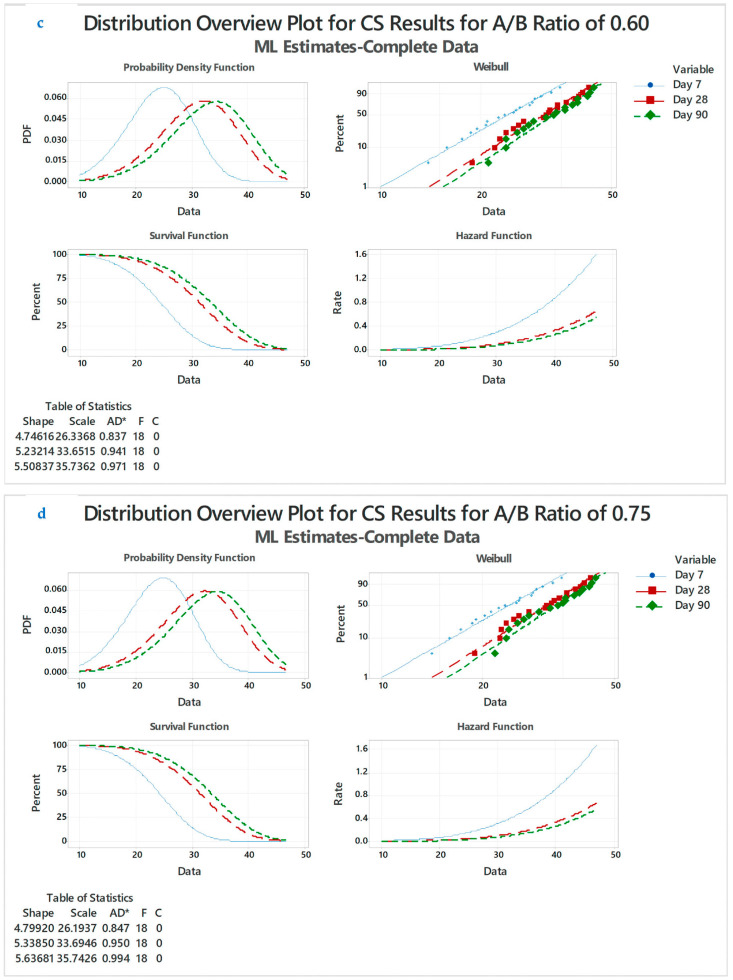
(**a**). DO curve plot for CS results for A/B ratio of 0.30; (**b**). DO curve plot for CS results for A/B ratio of 0.45; (**c**) DO curve plot for CS results for A/B ratio of 0.60, and (**d**) DO curve plot for CS results for A/B ratio of 0.75.

For the distribution study of A/B ratios examined for 7, 28, and 90 days, three distinct types of mixtures were used. The DO curve for CS findings is shown in Figure 6a–d. Mini tab software was used to evaluate the DO curve. For an A/B ratio of 0.30, NDT the results of CS values tested for 7, 28, and 90 days was taken for plot of DO curve probability density function (PDF), Weibull, survival and hazard function, the curves of which were represented in DO plot in Figure 6a. The PDF curve for GPC specimens, when tested for day 7, shows that the PDF of 0.010 is higher compared with the specimens tested for 28 and 90 days. This PDF value indicates that as the days progress, the density of GPC and strength start to reduce with a 0.010 PDF factor. The PDF curve for 28 and 90 days shows overlap and 90 days curve is slightly high compared with day 28. The 28- and 90-day results show almost identical characteristics. 

Weibull plot for an A/B ratio of 0.30 shows that specimens cast for 28 and 90 days show linear ship. The Weibull indicates that the factors of Anderson-darling for day 7, 28, and 90 are 0.830, 0.938, and 0.983, respectively (Figure 6a). The curves are close together, with one end overlapping and other end with a slight deviation. Survival and hazard function curves also indicate that the 7 days curve has different distribution characteristics compared with day 28 and 90. GPC specimens cast with the same working conditions and factors such as CS, density, temperature exposure, and A/B ratios will change according to testing days [38,46,47].

#### 4.2.1. DO Curve for A/B:0.45

For an A/B ratio of 0.45, the NDT results of the CS values tested for 7, 28, and 90 days were taken for plot of DO curve probability density function (PDF), Weibull, survival and hazard function, the curves of which were represented in DO plot in Figure 6b. The PDF curve for the GPC specimens when tested for 7 days shows that the PDF of 0.015 is higher compared with the specimens tested for 28 and 90 days. This PDF value indicates that, as the days progress, the density of GPC and strength start to reduce with a 0.015 PDF factor. The PDF curve for 28 and 90 days shows overlap and the 90 days curve is slightly high compared with the 28 days curve. The 28 and 90 days results show almost identical characteristics. Weibull plot for an A/B ratio of 0.45 shows that specimens cast for 28 and 90 days shows linear ship. The Weibull indicates that the factors of Anderson-darling for days 7, 28, and 90 are 0.825, 0.972, and 1.032, respectively. The curves are close together with one end overlapping and the other end with a slight deviation. The survival and hazard function curves also indicate that the 7 days curve has different distribution characteristics compared with day 28 and 90. 

#### 4.2.2. DO Curve for A/B:0.60

For an A/B ratio of 0.60, the NDT results of the CS values tested for 7, 28, and 90 days was taken for plot of DO curve probability density function (PDF), Weibull, survival and hazard function, the curves of which were represented in DO plot in Figure 6c. The PDF curve for the GPC specimens when tested for 7 days shows that the PDF of 0.010 is higher compared with the specimens tested for 28 and 90 days. This PDF value indicates that, as the days progress, the density of GPC and strength start to reduce with a 0.010 PDF factor. The PDF curve for 28 and 90 days shows overlap and the 90 days curve is slightly high compared with days 28 curve. The 28 and 90 days results show almost identical characteristics. The Weibull plot for an A/B ratio of 0.30 shows that specimens cast for 28 and 90 days show linear ship. The Weibull indicates that the factors of Anderson-darling for days 7, 28, and 90 are 0.837, 0.941, and 0.971, respectively. The curves are close together with one end overlapping and other end with a slight deviation. The survival and hazard function curves also indicate that the 7 days curve has different distribution characteristics compared with day 28 and 90. The GPC specimens cast with the same working conditions and factors likes CS, density, temperature exposure, and A/B ratios will change according to testing days [29,39,46,48]. 

#### 4.2.3. DO Curve for A/B:0.75

For an A/B ratio of 0.75, the NDT results of the CS values tested for 7, 28, and 90 days were taken for plot of DO curve probability density function (PDF), Weibull, survival, and hazard function, the curves of which were represented in DO plot in Figure 6d. The PDF curve for GPC specimens when tested for 7 days shows that the PDF of 0.010 is higher compared with the specimens tested for 28 and 90 days. This PDF value indicates that, as the days progress, the density of GPC and strength start to reduce with a 0.010 PDF factor. The PDF curve for 28 and 90 days shows an overlap and the 90 days curve is slightly high compared with the 28 days curve. The 28 and 90 days results show almost identical characteristics. The Weibull plot for an A/B ratio of 0.30 shows that the specimens cast for 28 and 90 days show linear ship. The Weibull indicates that the factors of Anderson-darling for days 7, 28, and 90 are 0.847, 0.950, and 0.994, respectively. The curves are close together with one end overlapping and other end with a slight deviation. The survival and hazard function curves also indicate that the 7 days curve has different distribution characteristics compared with days 28 and 90 [49,50,51].

### 4.3. Empirical Commutative Distribution Factor (CDF)

**Figure 7:**
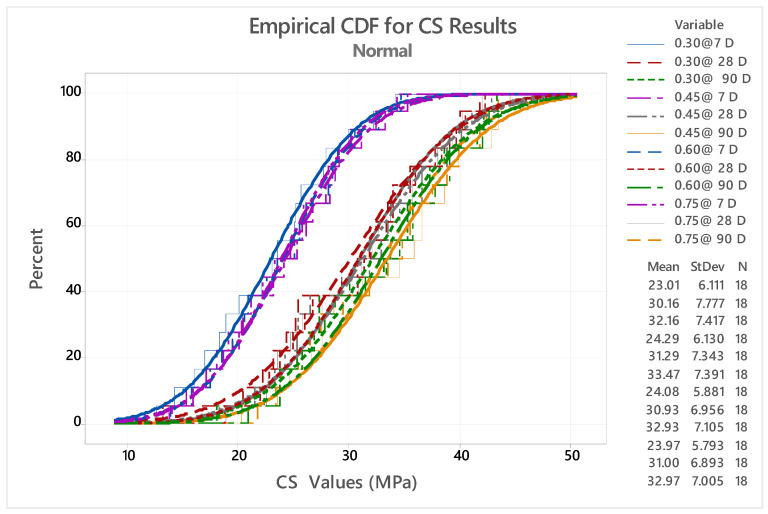
Commutative distribution factor (CDF) curve for CS results.

A total of 16 different types of mixes were taken into account for the CDF analysis for A/B ratios of 0.30, 0.45, 0.60, and 0.75 for 7, 28, and 90 days. Figure 7 shows the CDF curve for CS results. The CDF curve was analyzed using mini tab software. It is seen that 0.30 at 7 D, 0.45 at 7 D, 0.60 at 7 D and 0.75 at 7 D shows almost identical characteristic values to the GPC. The CS results for all A/B ratios for day 7 exhibit similar characteristics such as setting time and strength development. The GPC specimens tested at 28 and 90 days have similar characteristics, due to the GPC specimen exposure to 60 and 70 °C temperatures and the greater rate of geopolymerization results higher than the CS results [52,53,54,55,56].

### 4.4. Node CART for C1 vs. C2 to C10

**Figure 8:**
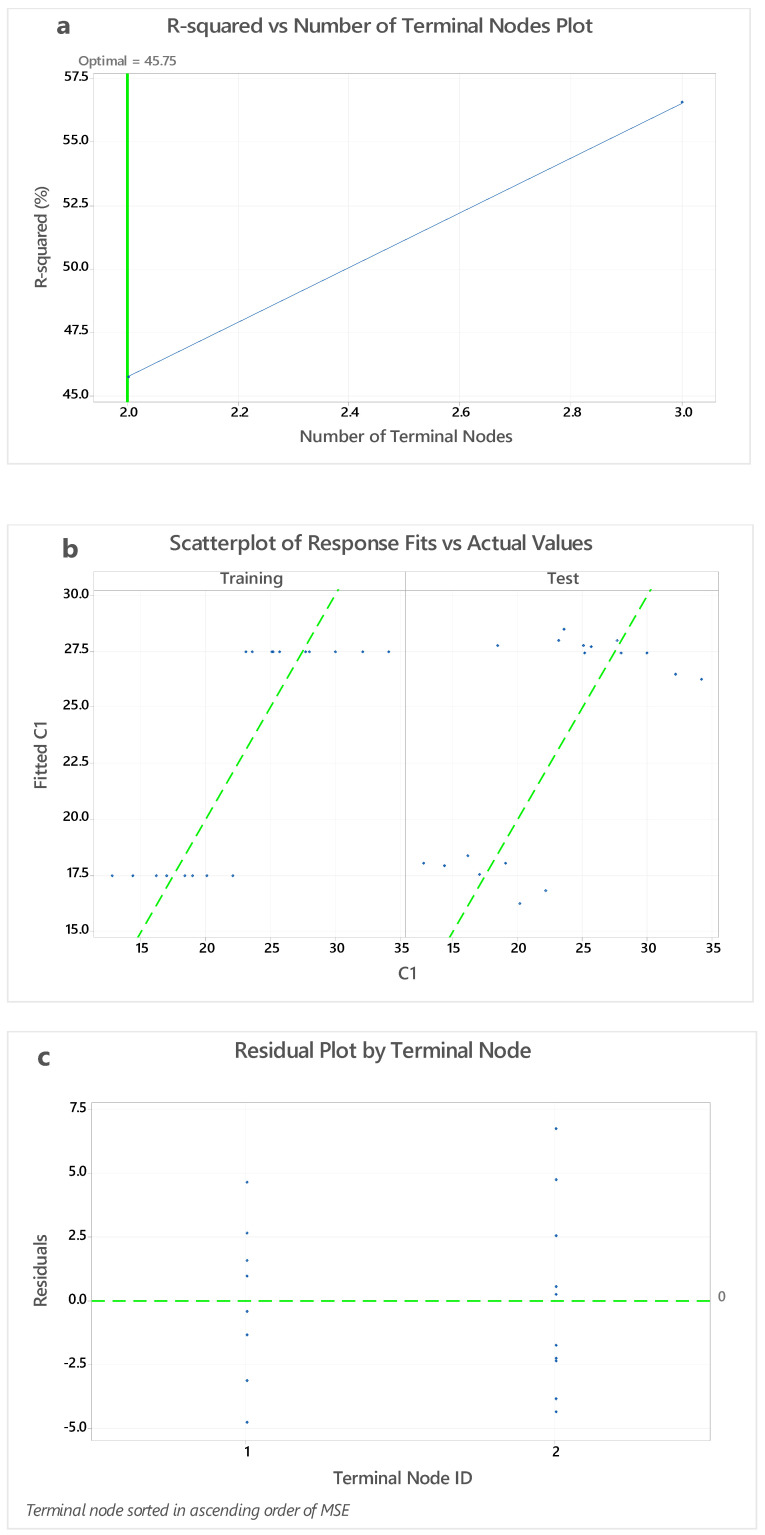
(**a**) Variation of R-squared and number of terminal nodes, (**b**) variation of scatterplot of response fits vs. actual values, and (**c**) variation residual plot by terminal node.

For the Node CART study of the A/B ratios examined for 7, 28, and 90 days, 16 distinct types of mixtures were used. Mini tab software was used to evaluate the DO curve. The node split technique was used for analysis the optimal tree for terminal node plot and the standard R-squared was carried out using a model validation of 10-fold cross-validation. The node CART for regression C1 versus C2, C3, C4, C5, C6, C7, C9, C8, C10, C11, C12 was considered for analysis (Figure 8a). A total of 16 different types of mixes used in the CDF analysis from the same specimens were taken into account for carrying node cart analysis by naming them as C1 to C10 in the same sequence (Figure 8c). C1 specimens, i.e., 0.30 at 7 D was taken as optimal line and C2 to C11 CS values were taken as terminal nodes. C1 vs. C2 to C11 shows a linear relationship. Figure 8b shows variation of scatterplot of response fits vs. actual values. With an optimum value of 45.75 with standard deviation of 6.11092, R-squared, training value was obtained as 70.25% and root mean squared error (RMSE) as 3.2390. The mean absolute percentage of error (MAPE) was 0.1247 [57,58,59,60]. 

The relationship between curing temperature and A/B ratio is a significant component that determines the strength of GPM. Because GGBS includes CaO, it leads to stable CS without much impact from the morality of the NaOH solution, and as a result, the CS is raised [39,61]. The greatest CS is seen for the A/B ratios of 0.45; beyond this ratio, the strength begins to decline. Excessive curing time resulted in a significant loss of strength. The evaporation of the liquid content in the mixture before to the end of the reaction time, as well as a rise in silica coagulation, were the main causes. The 7-day curve has a substantially separate distribution characteristic to the 28-day and 90-day curves, as seen by the terminal node [62,63].

## 5. Conclusions

The effect of temperature on the compressive strength parameter using destructive and non-destructive testing was analyzed. Some important observations were reported in this work.

The A/B ratio plays a vital role in strength development; an A/B ratio of 0.45 shows maximum compressive strength when the specimens are exposed to a curing temperature of 70 °C Furthermore, the A/B ratio of 0.45, under a 70 °C temperature, perforances of GPC reduces due to the evaporation of the liquid content in the mixture prior to the completion of the reaction duration, and even increases in silica coagulation.

- Appropriate methods need to be selected for the assessment of the strength properties of GPC. With the NDT approach, there a variation of results was observed compared with DT. For the non-destructive tests, the results showed almost 10% less compressive strength (CS) achieved compared with the destructive test.
- The probability and distribution overview shows a linear relationship between the compressive strength and an A/B ratio up to a specific point, i.e., an A/B ratio of 0.45 at 70 °C. The 7-day curve has a substantially separate distribution characteristic than the 28-day and 90-day curves, as seen by the survival and hazard function curves.
- The probability plot results in the range of 0.96–0.98, indicating that the CS results when tested at 7, 28, and 90 days vary linearly, with a 96 to 98% accuracy compared with reference mix.

## Figures and Tables

**Table 1:** Chemical composition of GGBS.

Composition Binder	SiO_2_	Al_2_O_3_	CaO	MgO	K_2_O	Fe_2_O_3_	Na_2_O	SO_3_	Others	LOI
GGBS (%)	34.80	15.78	36.81	7.09	0.44	0.38	0.27	2.53	-	1.50

**Table 2:** Physical properties of ingredients of GPC.

Materials	GGBS	Aggregate	R. Sand
Physical Properties			
Specific gravity	2.88	2.8	2.6
Zone	-	-	II
Fineness modulus	-	7.0	3.0
Silt content (%)	-	-	4
Blain Air Permeability (m^2^/kg)	400		

**Table 3:** Mix proportion of GPC.

Mix ID	GGBS %	Fine Aggregate (kg/m^3^)	Coarse Aggregate (kg/m^3^)	Alkali/ Binder Ratio	Alkaline Solution		Temperature ℃	Duration of Exposure of Temperature in Hours
					NaOH (kg/m^3^)	Na_2_SiO_3_ (kg/m^3^)		
G0	100	600	1300	0.30	14.66	52.4	50, 60, and 70	0, 2, 4, 6, 8, 10
G1	100	600	1300	0.30	14.66	52.4		
G2	100	600	1300	0.30	14.66	52.4		
G3	100	600	1300	0.45	14.66	52.4		
G4	100	600	1300	0.45	14.66	52.4		
G5	100	600	1300	0.45	14.66	52.4		
G6	100	600	1300	0.60	14.66	52.4		
G7	100	600	1300	0.60	14.66	52.4		
G8	100	600	1300	0.60	14.66	52.4		
G9	100	600	1300	0.75	14.66	52.4		
G10	100	600	1300	0.75	14.66	52.4		
G11	100	600	1300	0.75	14.66	52.4		

**Table 4:** DT results for various temperature and A/B ratios.

Parameters			Duration of Temperature/Hour					
Temperature/°C	A/B Ratio	Testing/Days	0 h	2 h	4 h	6 h	8 h	10 h
			Compressive Strength in/MPa					
50	0.3	7	14.8	16.1	19.1	21.58	25.9	27.89
		28	18.5	23	27	30.1	33.8	37.99
		90	20.1	23.41	28.95	31.58	35.87	40.29
	0.45	7	15.8	17.4	20.2	23.5	27.7	30.3
		28	20.3	24.5	29.3	32.4	36.5	39.6
		90	22.4	25.6	31.6	33.3	37.9	41.9
	0.6	7	15.2	16.8	19.6	22.9	27.1	29.7
		28	19.8	24	28.8	31.84	35.94	39.04
		90	21.62	24.82	30.82	32.52	37.12	41.12
	0.75	7	14.9	16.5	19.3	22.6	26.8	29.4
		28	19.55	23.75	28.55	31.59	35.69	38.79
		90	21.28	24.52	30.52	32.22	36.82	40.78
60	0.3	7	16.98	20.08	23.08	25.68	27.98	32.18
		28	22.22	24.42	30.82	33.52	37.82	40.02
		90	23.35	26.35	33.35	35.35	38.65	42.25
	0.45	7	18.1	21.2	24.2	26.8	29.1	33.3
		28	23.3	25.5	31.9	34.6	38.9	41.1
		90	24.6	27.6	34.6	36.6	39.9	43.5
	0.6	7	17.5	20.6	23.6	26.2	28.5	32.7
		28	22.8	25	31.4	34.04	38.34	40.54
		90	23.82	26.82	33.82	35.82	39.12	42.72
	0.75	7	17.2	20.3	23.3	25.9	28.2	32.4
		28	22.55	24.75	31.15	33.79	38.09	40.29
		90	23.48	26.52	33.52	35.52	38.82	42.38
70	0.3	7	18.98	22.08	25.08	27.68	29.98	34.18
		28	23.22	25.42	31.82	35.52	39.12	41.82
		90	25.35	27.35	35.35	37.35	41.65	43.35
	0.45	7	20.1	23.2	26.2	28.8	31.1	35.3
		28	24.3	26.5	32.9	36.6	40.2	42.9
		90	26.6	28.6	36.6	38.6	42.9	44.6
	0.6	7	19.5	22.6	25.6	28.2	30.5	34.7
		28	23.8	26	32.4	36.04	39.64	42.34
		90	25.82	27.82	35.82	37.82	42.12	43.82
	0.75	7	19.2	22.3	25.3	27.9	30.2	34.4
		28	23.55	25.75	32.15	35.79	39.39	42.09
		90	25.48	27.52	35.52	37.52	41.82	43.48

## Data Availability

The data that support the findings of this study are available on request from the corresponding author [SVG].

## Abbreviations

A/B	Alkali/binder ratio
Al	Alumina
ASTM	American Society for Testing and Materials
Al_2_O_3_	Aluminum oxide
CA	Calcium
CASH	Calcium-alumina-silica-hydrate
CE	Carbon emission
CS	Compressive strength
CTM	Compression testing machine
DCS	Destructive compressive strength
EE	Embodied energy
FA	Fly ash
GGBS	Ground granulated blast-furnace slag
GPB	Geopolymer brick
IS	Indian standard
K	Potassium
Kg	Kilogram
MPa	Mega Pascal
MJ	Mega Joule
Na	Sodium
Na_2_SiO_3_	Sodium silicate
NaOH	Sodium hydroxide
NASH	Sodium-alumina-silica-hydrate
NDT	Non-destructive testing
O	Oxygen
RHA	Rich husk ash
Si	Silica
SiO_2_	Silicon oxide
SiO_4_	Silicon oxygen tetrahedron
WA	Water absorption
°C	Degrees Celsius
